# Evolution of Singlet Oxygen by Activating Peroxydisulfate and Peroxymonosulfate: A Review

**DOI:** 10.3390/ijerph18073344

**Published:** 2021-03-24

**Authors:** Guangfeng Xiao, Tiantian Xu, Muhammad Faheem, Yanxing Xi, Ting Zhou, Haseeb Tufail Moryani, Jianguo Bao, Jiangkun Du

**Affiliations:** School of Environmental Studies, China University of Geosciences, Wuhan 430074, China; doujiang1226@126.com (G.X.); xutiantian202102@163.com (T.X.); Faheem@cug.edu.cn (M.F.); xiyanxing525@163.com (Y.X.); 18202721355@163.com (T.Z.); haseebmoriani@cug.edu.cn (H.T.M.); bjianguo@cug.edu.cn (J.B.)

**Keywords:** singlet oxygen, peroxydisulfate, peroxymonosulfate, activation, organic contaminants, degradation

## Abstract

Advanced oxidation processes (AOPs) based on peroxydisulfate (PDS) or peroxymonosulfate (PMS) activation have attracted much research attention in the last decade for the degradation of recalcitrant organic contaminants. Sulfate (SO_4_^•−^) and hydroxyl (^•^OH) radicals are most frequently generated from catalytic PDS/PMS decomposition by thermal, base, irradiation, transition metals and carbon materials. In addition, increasingly more recent studies have reported the involvement of singlet oxygen (^1^O_2_) during PDS/PMS-based AOPs. Typically, ^1^O_2_ can be produced either along with SO_4_^•−^ and ^•^OH or discovered as the dominant reactive oxygen species (ROSs) for pollutants degradation. This paper reviews recent advances in ^1^O_2_ generation during PDS/PMS activation. First, it introduces the basic chemistry of ^1^O_2_, its oxidation properties and detection methodologies. Furthermore, it elaborates different activation strategies/techniques, including homogeneous and heterogeneous systems, and discusses the possible reaction mechanisms to give an overview of the principle of ^1^O_2_ production by activating PDS/PMS. Moreover, although ^1^O_2_ has shown promising features such as high degradation selectivity and anti-interference capability, its production pathways and mechanisms remain controversial in the present literatures. Therefore, this study identifies the research gaps and proposes future perspectives in the aspects of novel catalysts and related mechanisms.

## 1. Introduction

Nowadays the wide occurrence of emerging organic and refractory pollutants in the soil and aquatic environment has raised much concern about water and food security. Advanced oxidation processes (AOPs) are feasible options to remove these undesirable pollutants by producing reactive oxidizing species (ROSs), such as hydroxyl radicals (^•^OH, E^0^ = 2.8 V) and sulfate radicals (SO_4_^•−^, E^0^ = 2.5 − 3.1 V) [1,2]. In the past two decades, AOPs using peroxydisulfate (PDS, S_2_O_8_^2^^−^) and peroxymonosulfate (PMS, HSO_5_^−^) as oxidants have attracted much attention for the non-selective degradation of a wide range of pollutants. Because PDS and PMS are solid powders, they can be easily delivered. Compared to H_2_O_2_, the anions of PDS/PMS can remain stable in water much longer until being properly activated. In addition, PMS and PDS-based AOPs can be smoothly carried out over a broad solution pH range from acid to alkaline while H_2_O_2_-based Fenton processes require strict acidic conditions. Typically, PDS and PMS should be properly activated by breaking the O-O bond with the aid of ultraviolet, heat, alkali or metallic catalysts and so on. SO_4_^•−^ and ^•^OH are commonly produced as the main ROSs [3]. Despite how promising both SO_4_^•−^ and ^•^OH are for degrading organic contaminants, their widespread application to wastewater has been limited by the presence of radical quenchers, such as some inorganic anions and naturally-occurring organic compounds [4,5]. However, many recent articles also indicate that singlet oxygen (^1^O_2_, a non-radical reactive oxidizing species) can be produced for pollutants degradation via a non-radical process instead of radical attacking pathways [6]. Compared to SO_4_^•−^ and ^•^OH, ^1^O_2_ shows higher selectivity to electron-rich organics because of its electrophilic nature [7,8,9]. This advantage is conducive to the degradation of micropollutants, such as pharmaceuticals and endocrine-disrupting compounds (EDCs), in the coexistence of salinity and other organic matter.

The other well-known method for ^1^O_2_ generation is photosensitized excitation of molecular oxygen. Photosensitizers including dyes, porphyrins, transition metal complexes and semiconductors act as the medium by transferring the light energy to ground dioxygen [10]. Other approaches to form ^1^O_2_ involve the use of chemicals like potassium perchromate [11], ozonide [12], H_2_O_2_-hypochlorous [13], periodate [14], and bismuth oxides [15]. But these processes have to use toxic sensitizers or consume too much bismuth precursors. As an alternative, activating PDS/PMS is a feasible and green chemical process for ^1^O_2_ production.

Current studies suggest that catalyst is influential to regulate the oxidizing species formed during PDS/PMS-based AOPs. For example, ^1^O_2_ has been frequently found as the primary reactive species by using carbonaceous catalysts in PDS and PMS activation [16], but SO_4_^•−^ and ^•^OH are usually found to be the dominant species when PDS/PMS are activated by homogeneous heat, UV, and transition metals [3,17]. The difference between PDS and PMS leads to different activation pathways. In particular, a study by Wang and others indicated that SO_4_^•−^ was mainly formed during MnO_2_-induced activation of PMS [18], but ^1^O_2_ was evolved when PMS was replaced by PDS [19]. Given the increasing interest in PDS/PMS, some recent works have reviewed the application of PDS/PMS-based AOPs for water treatment [2,3,4,17,20,21]. Most of these reviews focus on the generation of SO_4_^•−^, however, a comprehensive assessment of ^1^O_2_ formation during PDS/PMS activation is rarely reported.

The present study reviews recent progress of ^1^O_2_ formation in PDS/PMS activating systems. A brief introduction of the chemistry of ^1^O_2_, performance of various types of catalysts, and insights into the possible mechanisms are critically discussed here. Finally, the research barriers and future perspectives are summarized in the last part. This review will provide more guidance for future research of PDS/PMS activation for water purification.

## 2. Chemical Feature of ^1^O_2_ and Its Oxidation

**Figure 1:**
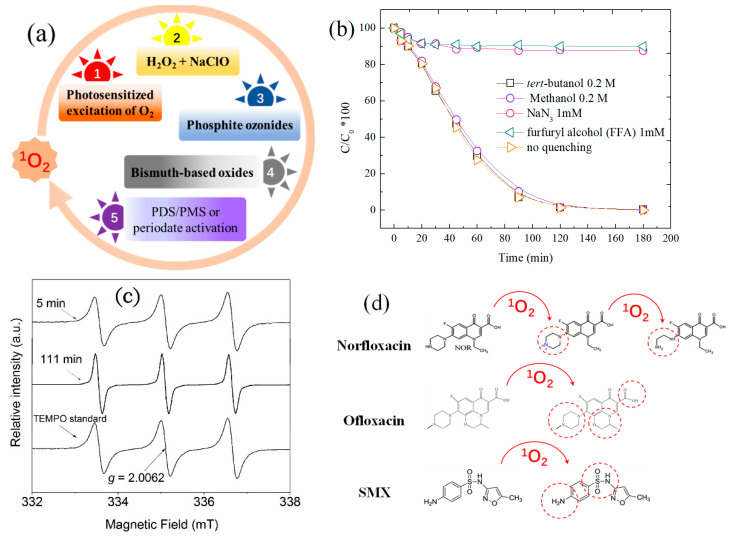
(**a**) Reported pathways of ^1^O_2_ evolution; (**b**) the quenching effect of ^1^O_2_ on phenol degradation with β-MnO_2_ (data reorganized from [19]); (**c**) EPR characteristic spectrum of TEMP-^1^O_2_ (figure reprinted from [31]). The EPR spectrometer settings were as follows: modulation frequency, 100 kHz; modulation width, 0.079 mT; scanning field, 335 ± 10 mT; amplitude: 2–500; time constant, 0.1–0.3 s; sweep time, 4 min; microwave power, 4 mW; and microwave frequency, 9.41 GHz.; (**d**) oxidation pathways of some emerging contaminants by ^1^O_2_ [8,34,38], reprinted with permission from Elsevier.

Singlet oxygen (^1^O_2_) is the excited molecular oxygen, and broadly refers to two low-lying states oxygen species O_2_ (^1^Δ_g_) and O_2_ (^1^Σ_g_^+^). Due to its short lifetime (10^−12^ s), O_2_ (^1^Σ_g_^+^) is easily converted to lower excited O_2_ (^1^Δ_g_) (lifetime 10^−3^–10^−6^ s) [22]. Herzberg first defined this excited oxygen with higher energy as ^1^O_2_, but its importance was not recognized until 1964 when scientists established its role in chemical oxidation [23]. ^1^O_2_ is a non-radical species with energy of 94.2 kJ per mole above ground-state molecular oxygen. As a mild oxidizing species, its standard redox potential (E^0^ = 1.52 V) is significantly lower than that of ^•^OH (E^0^ = 2.8 V) and SO_4_^•−^ (E^0^ = 2.5 − 3.1 V) [24,25]. The generation of ^1^O_2_ has been reported in a relatively broad pH range by various approaches (Figure 1a) including: (1) photosensitized excitation of triplet dioxygen [10,26]; (2) reaction of H_2_O_2_ with NaClO [13]; (3) released from phosphite ozonides, bismuth oxides and some organic peroxides [12,15]; (4) proper activation of inorganic oxidants, such as PDS/PMS and periodate [7,14,27].

Due to its specific characteristics, ^1^O_2_ has been applied in organic synthesis by selective oxidation [28], aqueous pollutant degradation [7], pathogenic bacteria inactivation [29] and medical cancer therapy [30]. The involvement of ^1^O_2_ in these fields can be monitored by mainly three approaches including chemical probing tests, electron paramagnetic resonance (EPR) spectrometry and also chemiluminescence detection. Sodium azide (NaN_3_) and furfuryl alcohol (FFA) are two popular ^1^O_2_ quenchers. The reaction rate of ^1^O_2_ with NaN_3_ and FFA have been tested to be 1×10^9^ M^−^^1^•s^−^^1^ and 1.2×10^8^ M^−^^1^•s^−^^1^, respectively [19]. However, ^1^O_2_ is not sensitive to alcohols, such as methanol, ethanol and tert-butanol, which are normally used for SO_4_^•−^ and ^•^OH quenching. Therefore, the presence of ^1^O_2_ can be identified by using different chemical quenchers as indicated in Figure 1b. Moreover, ^1^O_2_ can be captured by a spin trapping agent, 2,2,6,6-tetramethyl-4-piperidinol (TEMP), to form 2,2,6,6-tetramethyl-4-piperidone-N-oxyl (TEMPO) which could be detected by EPR with an intensive 1:1:1 signal (Figure 1c) [31]. In addition, direct or indirect chemiluminescence is also effective for the detection of ^1^O_2_. For example, ^1^O_2_ could be measured by solid-state near infrared spectroscopy at 1270 nm, which is the radiative photon released from ^1^O_2_ because of its intrinsic energy difference compared to ground state oxygen [32]. Fluorescence detection is another helpful technique for ^1^O_2_ detection. Yuan et al. reported a conjugated probe consisting of a photosensitizer and a fluorogenic dye that were linked by aminoacrylate (AA) [33]. By reacting with ^1^O_2_, the AA linker was cleaved and the probe divided to yield green fluorescence.

As an excited oxygen species, ^1^O_2_ shows high affinity for electrophilic oxidation of electron-rich compounds with unsaturated C=C bonds as well as sulfide and amine groups. This natural utility endows ^1^O_2_ with the capability to selectively degrade pharmaceutical pollutants. Zhou et al. investigated the degradation of sulfamethoxazole by ^1^O_2_, and found that sulfanilic group rather than isoxazole ring was the attack site [34]. Electron transfer and formation of endoperoxide intermediate were possible pathways involved in the ^1^O_2_-induced sulfamethoxazole oxidation, specifically resulting in hydroxylation of the aniline ring, amine oxidation and oxidative coupling of the two intermediates (Figure 1d). Gao et al. discovered that the piperazinyl, oxazinyl and carboxylic substituents of ofloxacin were prone to be attacked by ^1^O_2_ in the LaBO_3_/PMS reaction system [8]. Hydroxyl addition, C-N cleavage, demethylation and decarboxylation were possible degradation pathways for ^1^O_2_-mediated oxidation of ofloxacin (Figure 1d). Liu et al. found that the olefinic bonds and C-N bonds of carbamazepine were susceptible to electrophilic ^1^O_2_ oxidation during PMS activation with N-doped carbon fiber aerogel [9].

**Figure 2:**
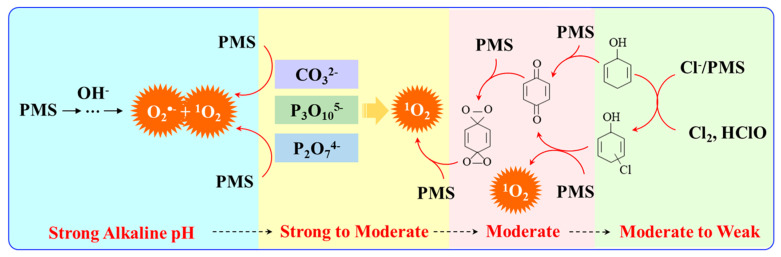
Evolution of ^1^O_2_ in homogeneous activation systems requiring different alkaline conditions.

An earlier study indicated that the interaction between chlorophenols and PMS would produce SO_4_^•−^, ^•^OH as well as ^1^O_2_, which then react with phenols or chlorophenols to generate hydroperoxides, and the later intermediate could dehydrate with the formation of p-benzoquinone (Figure 2) [35]. Under alkaline conditions, the reaction rate of ^1^O_2_ toward dissociated chlorophenols is in the range of 1.60 × 10^8^ M^−^^1^•s^−^^1^ to 1.93 × 10^8^ M^−^^1^•s^−^^1^, which can be slightly influenced by substitutes. In addition, organic sulfides and disulfides were reported to be oxidized to sulfoxides and thiolsulfinates, respectively [10]. Charge transfer and radical propagation were suggested as the two main mechanisms for ^1^O_2_ oxidation. However, it should be noted that ^1^O_2_ is not as strong as radicals of SO_4_^•−^ and ^•^OH for the depletion of wastewater COD or TOC [36]. Given its anti-interference and selective feature, ^1^O_2_ is frequently studied for tumor cell inactivation via protein oxidation [37].

## 3. Evolution of ^1^O_2_ by Activating PDS/PMS in Homogenous Systems

Although thermo-, UV- and alkali- catalytic activation have proven to be effective for both PDS and PMS, most of these homogeneous systems degrade organic contaminants by forming free radicals, such as SO_4_^•−^ or ^•^OH. Instead, as indicated in Table 1, the evolution of ^1^O_2_ has been recently discovered when PMS was activated by quinones, phenols, alkali, etc.

Ketones, quinones, and phenols exist ubiquitously in water and soils. Studies have found that these hydrocarbons can activate PMS, and further suggested that ^1^O_2_ might be the main oxidizing species. In 1974, Montgomery discovered that the decomposition rate of PMS was substantially proportional to the amount of ketone at low range of concentrations [39]. Lange and Brauer further verified that ^1^O_2_ was formed during ketone-catalyzed PMS activation by using infrared phosphorescence [40]. Similar to ketones with carbonyl groups, Zhou et al. found that benzoquinone (BQ) could effectively activate PMS to degrade sulfamethoxazole, and the degradation rate increased as solution pH increased from 7 to 10 [41]. Radical trapping tests indicated that ^1^O_2_ rather than SO_4_^•^^−^ and ^•^OH was produced in the BQ/PMS system. The proposed mechanism for BQ-mediated activation of PMS, as illustrated in Figure 2, can be described by Equations (1)–(4). Firstly, PMS anions are added to the carbonyl group of BQ via nucleophilic attack and converted to peroxide adduct labeled as intermediate I as shown in Equation (1). Under alkaline conditions, intermediate I undergoes dehydrogenation and forms intermediate II (Equation (2)), which is further transformed into dioxirane (intermediate III, Equation (3)). Finally, the dioxirane reacts with ionized PMS at a stoichiometric ratio of 1:2, producing ^1^O_2_ and BQ again (Equation (4)). It is noteworthy that, as stated by Gallopo et al., dioxirane can be formed as a key intermediate during the reaction between PMS and ketones or BQ through ^18^O labeling and kinetic studies [42]. Zhang et al. further detected dioxirane and ^1^O_2_ by using droplet spray ionization mass spectrometry (DSI-MS) as well as oxygen isotope analysis [43].


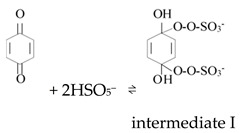
(1)


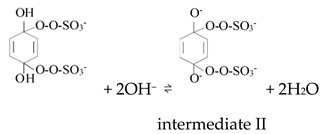
(2)


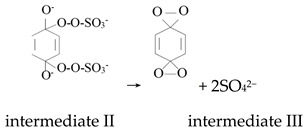
(3)


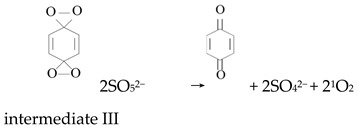
(4)

Apart from ketones and quinones, phenols are reactive enough to activate PMS because the phenolic group is easily oxidized to quinone byproducts. Zhou et al. found that PMS could be effectively activated to produce ^1^O_2_ by phenol at pH 8.5 and 10, in which phenol was oxidized to benzoquinone to promote PMS activation [34]. The chemical structure of phenols is a critical influencing factor for the overall reaction because the substituents and their positions on phenol could remarkably affect the yield of quinone intermediates. At acidic pH, phenols are poorly dissociated so that they can hardly form intramolecular complex with ionized PMS in such molecular state. Therefore, phenols are more likely to react with PMS in alkaline conditions [35]. In addition, it should be noted that the reaction between chloride and PMS at acidic pH would generate reactive chlorine species such as Cl_2_ and HClO, and further led to organic chlorination. But this undesirable side reaction was highly inhibited because ^1^O_2_ was mainly produced at alkali pH so that chloride showed negligible effect on phenol transformation [44].

In fact, alkali is a typical homogeneous PDS/PMS catalyst, and has been employed for in situ field remediation. The activation of PDS by alkali generates SO_4_^•−^, ^•^OH and superoxide (O_2_^•−^). Unlike PDS, the activation of PMS by alkali initiates non-radical attack for contaminants degradation. Qi et al. found that alkali can activate PMS at ambient temperature to generate O_2_^•−^ and ^1^O_2_ for oxidative degradation of acid orange 7 (AO7) [45]. At lower NaOH concentrations (0.6–1.0 mM), both O_2_^•−^ and ^1^O_2_ were formed, but only O_2_^•−^ was generated at higher doses of NaOH (8.0 mM). It was proposed that O_2_^•−^ formed and then recombined to generate one ^1^O_2_ and one hydrogen peroxide. But under extremely high pH conditions, the transformation of O_2_^•−^ to ^1^O_2_ was suppressed when the pH was higher than the p*K*_a_ of O_2_^•−^ as indicated by Equation (5) [46]. It was worth noting that in the studies from Qi [45] and Lou [47], the addition of p-BQ completely inhibited the oxidation reaction in the PMS/base system. But Zhou’s report pointed out that p-BQ could effectively activate PMS to generate ^1^O_2_ under alkaline conditions [41]. Thus the role of p-BQ in PMS activation might be closely related to its dose and pH conditions:

2O_2_^•^^−^ + 2H^+^ → H_2_O_2_ + ^1^O_2_ (5)

Base activation normally requires continuous addition of alkali to maintain desirable pH value. Instead, some studies have developed new effective homogeneous activators, such as chloride, carbonate and BO_2_^−^ as shown in Figure 2. For example, Lou et al. used polyphosphates to enhance the alkali activation of PMS, in which both O_2_^•−^ and ^1^O_2_ were detected as the main ROSs [47]. Polyphosphates are nucleophiles which promote the breakage of the peroxide O-O bond to speed up ^1^O_2_ formation. Like polyphosphate, BO_2_^−^ is also a nucleophile (Nu) which can bond with PMS to generate a NuOH^+^ intermediate, and then convert into ^1^O_2_ with excellent anti-interference performance [48]. Moreover, Nie et al. found that CO_3_^2^^−^ could activate PMS for the degradation of pharmaceuticals, phenols and dyes [49]. Species like ^1^O_2_ and O_2_^•−^ were identified, and the system showed good resistance toward the interference of Cl^−^, NO_2_^−^, HCO_3_^−^ and humic acid.

Chloride is another promoter for PMS activation under both acid and alkaline conditions. Wang et al. used the Cl^−^/PMS process for the treatment of coking wastewater concentrate, in which ^1^O_2_, hypochlorous acid and chlorine radicals were identified [50]. They discovered that the efficiency of the PMS/base/Cl^−^ system was vastly influenced by the dosage of Cl^−^ and NaOH [51]. The degradation of model pollutant was remarkably enhanced by Cl^−^ at low alkalinity, but inhibited when NaOH concentration was up to 2 mM. High alkalinity reduced the formation of organic halides in the PMS/base/Cl^−^ reaction system, which would be significant for the treatment of saline wastewater.

## 4. Evolution of ^1^O_2_ by Activating PDS/PMS with Metal-Free Carbon Catalysts

Metal-free carbon-based materials (MFCMs) are emerging heterogeneous catalysts for PDS/PMS-based oxidation processes in recent years. They have received much attention because of their attractive advantages over metal-based catalysts, such as less cost, no secondary pollution, and their chemical, thermal and mechanical stabilities. MFCMs including reduced graphene oxide (GO), carbon nanotubes (CNTs), nano-diamonds (NDs), carbon spheres (CS) and biochar in different sizes and specifications are investigated as potential PMS/PDS catalysts [16]. Unlike metal-catalyzed processes, organic pollutants can be removed via not only catalytic degradation but also adsorption by MFCMs.

**Figure 3:**
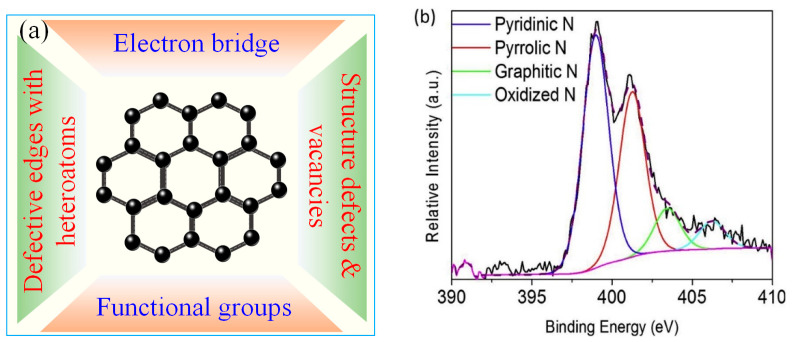
(**a**) The proposed active sites of MFCMs for ^1^O_2_ evolution via catalytic PDS/PMS activation. (**b**) The XPS spectra of N 1s for doping N element in different states [67], reprinted with permission from Elsevier.

To date, both radical and non-radical oxidations have been reported for MFCMs-mediated PMS/PDS AOPs (Table 2) [16,21]. The non-radical pathway is a potential route to resolve the influence of background organic and inorganic matters on the degradation of targeted pollutants. In a non-radical reaction system, MFCMs can serve as medium for electron transfer between PMS/PDS and organic pollutants. As listed in Figure 3a and Table 2, there are different active sites involved in activating PMS for ROSs generation. They include: (i) delocalized π-electrons (C-π) [52]; (ii) structural defects and vacancies [53]; (iii) heteroatoms bonded to carbon in the form of C=N-C and N-(C)_3_ [54]; and (iv) C=O and pyran-like oxygen functional groups [55] produced at vacancy defective edges (Equations (6)–(12)):

HSO_5_^−^ + C-π → SO_4_^•−^ + OH^−^ + C-π^+^ (6)

HSO_5_^−^ + C-π^+^ → SO_5_^•−^ + H^+^ + C-π (7)

HSO_5_^−^ + C=C=O → SO_4_^•−^ + OH^−^ + C=C-O^+^ (8)

HSO_5_^−^ + C=C-O^+^ → SO_5_^•−^ + H^+^ + C=C=O (9)

HSO_5_^−^ + C=N-C^+^ → SO_5_^•−^ + H^+^ + C-N-C (10)

SO_5_^•^^−^ + SO_5_^•^^−^ → 2SO_4_^2−^ + ^1^O_2_ (11)

[PDS/PMS] + [sp^2^/sp^3^-moieties] → non-radical / free-radical (12)

However, since most MFCMs are constructed with multiple structure and surface characteristics, the identification of the intrinsic active site remains difficult and controversial.

### 4.1. Carbon Nanotubes (CNTs)

CNTs are one dimensional quantum wires fabricated by rolling 2D graphite sheets. CNTs belong to the family of fullerene, and consist of sp^2^-hybridized atomic carbon in a hexagonal network. Based on their structural differences, there are single-walled CNTs (SWCNTs) and multi-walled CNTs (MWCNTs). With excellent adsorptive ability and electron conductivity, CNTs can act as good electron shuttle between organic pollutants and persulfate oxidants [56] so both CNTs-mediated electron transfer from organics to persulfate and ^1^O_2_ are responsible for a nonradical degradative route [57]. Yun et al. explored the role of PDS during its activation with nFe^0^ and CNTs [58]. Their results indicated that the radical oxidation process was dominant in the nFe^0^/PDS system but a non-radical mechanism was discovered in the CNTs/PDS system. Further chronoamperometric tests revealed that CNTs served as electron bridge for PDS and organic pollutants.

Noteworthily, the presence of different oxygen functional groups (OFGs) available on the surface of CNTs also play an important role because they directly influence the zeta potential of CNTs [59]. The removal of oxygen contents, especially the blockage of carboxylate group (-COOH) reduction to other carbonyl (C=O) and hydroxyl (-OH) groups through a annealing route is recommended to increase the zeta potential of CNTs. Consequently, this will facilitate the adsorptive uptake of PDS because of weaker electrostatic repulsion, and favor non-radical oxidation route of the targeted pollutants. PDS hydrolysis catalytically occurs when PDS complex with CNTs, and the surface nucleophilic C=O was found to be the crucial sites for the generation of O_2_^•−^ [60]. The produced O_2_^•−^ then recombines and is finally converted to ^1^O_2_ with the production of hydrogen peroxide as well.

In other cases, surface N-doped CNTs were also investigated compared to pristine material. Sun et al. reported that CNTs doped with 0.88 at.% of nitrogen could achieve a better efficiency (as high as 7-fold) than pristine CNTs for catalytic PMS oxidation of phenol, but showed decreased efficiency on PDS activation. Free sulfate radicals were discovered to be responsible for phenol degradation in the same study [61]. However, some researchers also obtained a contrasting result that non-radical oxidation was dominant in the N-CNTs/PMS system [62]. Because of greater electronegativity, it was postulated that the doped N would enhance the interaction between the carbon atoms and PMS, and thus boost electron transfer.

### 4.2. GO/rGO

Graphene oxide (GO) is a single-atomic layered carbon material laced with various oxygen-containing groups that fabricated by powerful oxidation of graphite. As a graphene-based material, GO and its reduced derivative rGO have shown great potential in the hydrophilic adsorption of organics and PMS/PDS catalysis, due to various structural defects, vacancies and C=O functional groups [53]. As indicated by density functional theory (DFT) calculations, vacancies and defective edges of rGO would prolong the O-O bond of PMS molecules, enhance adsorption and direct electron transfer, thus facilitate final break-up of O-O bond to initiate nonradical oxidation [63].

The increase of carbonyl groups and graphitization degree would create more vacancies and defects to enhance the catalytic performance of GO/rGO [27]. A simple but effective strategy is to modulate rGO by heteroatom doping [64]. Carbon doped with N is expected to possess more lattice defects for regulating the electronic structure, such as sp^2^-hybridized carbon skeletons [65,66]. Kang et al. reported that N-doping could significantly improve the activity of reduced graphene oxide (N-rGO) for PMS activation using urea as the nitrogen source [67]. It was found that pyridine N, pyrrole N, graphite N, and oxidized N in N-rGO catalyst accounted for 49.7%, 35.2%, 9.0%, and 5.9%, respectively (Figure 3b). ^•^OH, SO_4_^•−^ and ^1^O_2_ were potential active species for pollutant degradation. As previous reported, pyrrolic N sites were important to adsorb and activate PMS to form ^1^O_2_ [68]. Doping nitrogen into carbon matrix is not only beneficial to PMS adsorption due to the increase of surface basicity, but also facilitates electron transfer to the negatively charged PMS, thereby enhancing the catalytic activity.

Besides, the co-doping of other heteroatoms also results in synergistic increase in catalytic performance to generate ROSs. Sun et al. introduced sulfur and nitrogen element into rGO to synthesize a catalyst named i-rGO-NS [69]. The XPS characterizations suggested that the additional sulfur doping increased the content of graphite N (33.74%) and change the distribution of N atom in RGO. Sulfur dopants existed in the form of thiophene S and oxidized S. Compared to rGO, i-rGO-N and metal catalysts, i-rGO-NS showed better reactivity to PMS oxidation of methyl p-hydroxybenzoate (MP). Quenching tests and EPR results indicated that ^1^O_2_ was the dominant species but ^•^OH and SO_4_^•−^ scarcely contributed to MP removal. In general, pyrrole N and pyridine N could activate the π electrons of the sp^2^ carbon atoms on rGO, and induce the activation of PMS to produce SO_4_^•−^, while graphite N could promote the transfer of electrons to PMS to generate ^1^O_2_ by neighboring carbon atoms [70]. In addition, thiophene S could mediate the redistribution of charge density to promote the creation of ^1^O_2_ [71]. Chen et al. reported that the sole N-atom doping could interrupt the spin and charge dispersion of the uniform sp^2^-hybridized configuration, leading to graphene chemical inert. However, the co-doping of a second dopant B-atom would activate carbon atoms adjoining to the N-atom. This provided higher electron and spin density, which accelerated the PMS activation through non-radical mechanism [72].

In contrast, high temperature heating of carbon catalysts brings about challenges of poor material dispersion and hydrophilic-to-hydrophobic transformation. As an alternative solution, Zhang et al. anchored amino-functionalized mesoporous silica (NH_2_-MCM-41) to N-doped GO materials (NG) to improve the hydrophilicity of NG materials, greatly improving the performance on simultaneous adsorption and degradation of contaminants [73]. The introduction of amino groups realized the inversion of negative charge to positive charge, enhanced the electrostatic interaction between the surface of NG and phenolic pollutants, and facilitated the removal of pollutants. Additionally, amino groups would increase the electron transfer capacity of NG to promote catalytic activation [74]. The mesoporous channel of NH_2_-MCM-41/NG provided effective transport and reaction units for PMS, pollutants and also ^1^O_2_.

### 4.3. Biochar

Biochar is derived from biomass carbonization under an oxygen-free environment. Biochar is cheap and widely available, and functions as a way for carbon sequestration. In recent years, biochar has attracted significant attentions for PDS/PMS activation. Biomass type and pyrolysis temperature are important factors influencing the biochar structural features. For example, biochar obtained through high-temperature pyrolysis (800 °C) have shown structural oxygen defects, which acted as electron conductor moieties for molecular O_2_ activation via non-radical routes [75]. This finding provided a novel approach to obtain biochar with vacancy defects capable of catalytic pollutants degradation through a non-radical pathway. Moreover, Huang et al. has explored the role of ketone structure of sludge-derived biochar [76]. The formation of ^1^O_2_ was detected for the mineralization of BPA. It was deduced that creation of epoxy structure was a possible course to generate ^1^O_2_ for ketone-catalyzed PMS decomposition as presented in Equations (13)–(16):

BC-RCOR* + HSO_5_^−^ → BC-RCOHR*(O-O-SO_3_^−^) (13)

BC-RCOHR*(O-O-SO_3_^−^) + OH^−^ → BC-RCO^−^-R*(O-O-SO_3_^−^) + H_2_O (14)

BC-RCO^−^-R*(O-O-SO_3_^−^) → BC-RCOOR* + SO_4_^2^^−^ (15)

BC-RCOOR* + SO_5_^2^^−^ → BC-RCOR* + SO_4_^2^^−^ + ^1^O_2_ (16)

Additionally, a recent study revealed the role of doped N and S for the catalytic activity of modified biochar [77]. The N-doped biochar gave a positive while S-doping demonstrated a negative effect on biochar-catalyzed PMS activation for metolachlor degradation. It was suggested that N-doping would augment more positive charge of the neighboring C atoms to interact with negatively charged HSO_5_^−^ species. However, in the case of S-doping, there was insignificant charge transfer due to the disruption of charge redistribution, which referred to breakage of charge balance in covalent carbon electron system. The synergistic effect of the heteroatom N-doping and the prevailing structural defects of graphene both contributed to induce non-radical pathway for the catalytic PMS oxidation of phenol [78]. Yin et al. prepared N-doped sludge-derived biochar (SDBC) with similar Raman spectral characteristics to graphene oxide [79]. They discovered that SDBC could efficiently activate PDS for the removal of sulfamethoxazole through ^1^O_2_-mediated degradation, in which 94.6% of sulfamethoxazole (SMX) and 58% of TOC were removed after 180 min of reaction. Furthermore, a high value of I_d_/I_g_ of SDBC indicated abundant amount of defect sites inside the carbon layer structure which were possible catalytic sites.

Regarding nitrogen tailing, sewage sludge usually contains N from microbial cells and can be utilized to produce low-N doped sludge biochar. Mian et al. investigated the effectiveness of chemically treated sludge-based biochar for the degradation of organic dyes [80]. It was disclosed that pyridinic-N active sites were the main contributor for the catalytic degradation through non-radical pathway, while pyrrolic-N, activated C^(+)^ as well as surface area acted as active sites for the adsorptive uptake of the pollutants under consideration. Due to the remarkable role of doped N, some studies attempted to increase the N doping amount. Hu et al. doped nitrogen into sludge-derived biochar using urea as a supplementary N source [81]. BET tests and Raman spectroscopy unveiled that the addition of urea improved the specific surface area and the number of active sites for interaction with PMS. Compared to non-doped sludge biochar (C-700), the new N-doped catalyst NC-700 exhibited better activity to remove organic pollutants by synergistic effect of adsorption and catalytic PMS oxidation. The adsorption capacity of methylene blue (MB) on NC-700 reached 35.83 mg/g and the removal rate of MB in NC-700/PMS system was 98.7% after 20 min. The chemical quenching and EPR tests clearly supported that large amount of ^1^O_2_ but little ^•^OH and SO_4_^•−^ were produced in the reaction system, affirming the non-radical pathway induced by biochar.

### 4.4. Other MFCMs

Many other MFCMs in different dimensions and structure also have shown good reactivity for PDS/PMS activation. Graphited nanodiamond (G-ND) demonstrated superior activation for both PMS and PDS when compared with other metal-free catalysts such as graphene, CNTs, graphite, and fullerene. For example, G-ND showed excellent catalytic performance in persulfate system for the mineralization of phenolic compounds and pharmaceuticals through non-radical pathway [82]. It was deduced from different analysis that G-ND provided surface binding sites for both PDS and phenol molecule to a close proximity. In the formed charge transfer complex, phenol acted as an electron donor and PDS served an electron acceptor, while G-ND functioned as a facile electron transfer mediator channel. Moreover, no inhibition was observed in the existence of oxidant scavengers as well as unwanted natural organic matters. Additionally, the temperature effect on the proportion of graphitic natural carbon in the sp^2^/sp^3^ configurations of NDs has been investigated in detail [83]. It was revealed that higher annealing temperature (1100 °C) treatment provided more graphitic shell than the lower annealing temperature (900 °C). The NDs catalyst obtained at 1100 °C (S-ND-1100) contributed to non-radical oxidation route, but the NDs-based catalyst achieved at 900 °C provided radical-dominated oxidation route during PMS activation.

Typically, PDS and PMS were adsorbed and activated on a carbon surface [84]. Jiang et al. successfully developed a metal-free porous carbon aerogel (CA) through the hydrothermal carbonization route by using D-glucose, ammonium persulfate, and aniline [85]. The sp^2^-hybridized moieties available on CA surface would interact with PDS and dissociate the O-O bonds of PDS. Then the active complex acquired from the first stage initiated the oxidation of rhodamine B (RhB) directly via electron transfer mechanism without the generation of free radicals. In another study, both urea and NaHCO_3_ were used to functionalize chitosan-derived carbon nanosheets with graphene-like structures [86]. The as-obtained material reflected great potential for the oxidation of recalcitrant pollutants by activating PMS to produce ^1^O_2_ as the main ROSs.

## 5. Evolution of ^1^O_2_ by Activating PDS/PMS with Metal Catalysts and Their Composite

Transition metals and metal oxides, such as Co, Mn, Fe, Cu, are effective catalysts for activating PDS/PMS, normally without extra assistance of light and heat. The activation processes with transition metals highly rely on the interaction between PDS/PMS and active redox sites, during which ^•^OH and SO_4_^•−^ are typically produced as the primary oxidative species. However, thanks to the improvement of analytical techniques, some recent studies found that ^1^O_2_ can also be generated from multiple non-radical pathways in metal/PDS or metal/PMS system configurations, including PDS/PMS self-decomposition, recombination of O_2_^•−^, and the mutual effect between catalysts and PDS/PMS [87,88]. Interestingly, these processes could take place either simultaneously or coupled with radical oxidations as illustrated in Table 3 and Table 4, which summarize the ^1^O_2_ evolution by activating PDS/PMS via heterogeneous transition metals.

### 5.1. Iron-Based Catalysts

Iron-based materials (e.g., zero-valent iron, Fe_3_O_4_) are widely used in AOPs because they are cheap and environmental-friendly. In general, the PMS/PDS activation with iron-based materials is accompanied by transformation from Fe(II) to Fe(III) and the generation of ^•^OH and SO_4_^•−^ (Equations (17)–(19)) [89]. Therefore, the amount of structural Fe(II) is a critical factor for catalytic PMS/PDS oxidation of organic pollutants:

Fe^2+^ + HSO_5_^−^ → Fe^3+^ + SO_4_^•−^ + OH^−^ (17)

Fe^2+^ + S_2_O_8_^2−^ → Fe^3+^ + SO_4_^•−^ + SO_4_^2−^ (18)

SO_4_^•−^ + H_2_O → SO_4_^2−^ + ^•^OH + H^+^ (19)

Yet non-radical oxidation is rarely reported for the Fe^0^/PMS or Fe^0^/PDS systems. The involvement of non-radical process can be observed under some specific conditions. In the study reported by Li et al., the spike of Cu^2+^ could enhance efficiency of the Fe^0^/PMS system by producing ^1^O_2_, O_2_^•−^ and also ^•^OH [90]. Similarly, the corrosion of Fe^0^ produces Fe^2+^ to activate PMS to form ^•^OH. However, the process could be interfered by Cu^2+^ spiking. Cu^2+^ would transform to Cu^0^, Cu^+^ and Cu_2_O by reacting with Fe^0^. The newly formed surface composite layer mediates PMS decomposition to a new pathway with production of ^1^O_2_ and O_2_^•−^. Yang et al. obtained the same results that ^1^O_2_ and O_2_^•−^ were dominant species and coexisted with ^•^OH in the Fe^0^-montmorillonite/PMS system [91]. Montmorillonite would alter the Fe^0^ surface oxidation layer which may affect the activation process [92].

**Figure 4:**
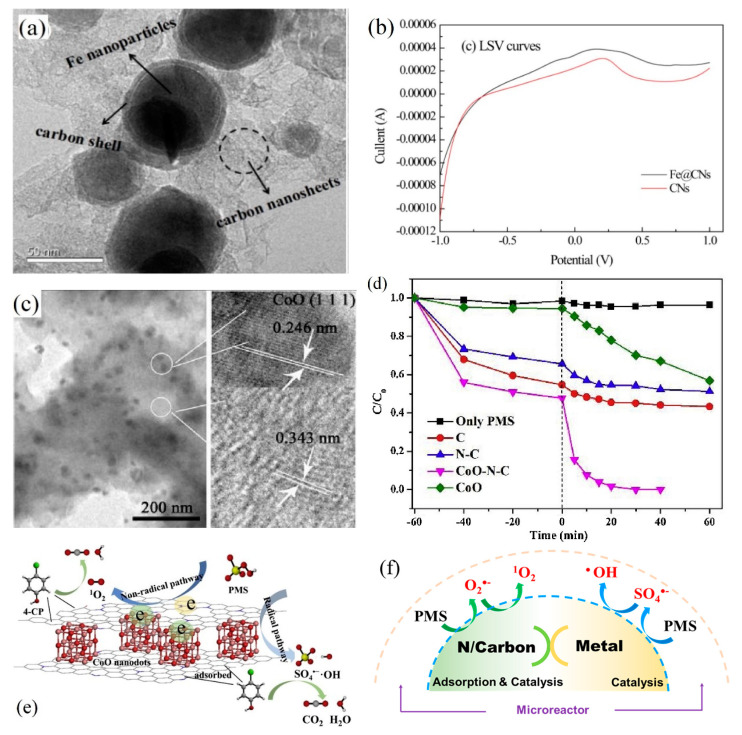
(**a**) TEM images of core-shell structure and (**b**) LSV curves of Fe@CNs [94], reprinted with permission from Elsevier; (**c**) TEM image of CoO nanodots distribution inside the carbon layers [102], reprinted with permission from Elsevier; (**d**) performance of CoO-N-C composite in comparison with other catalyst; (**e**) its catalytic mechanism [102], reprinted with permission; (**f**) schematic diagram of multi-ROSs generation in PMS/PDS activation systems with carbon/metal composite catalysts.

Another common iron catalyst is the nanoscale Fe_3_O_4_ which exhibits better stability than zero valent iron. The catalysis ability of Fe_3_O_4_ relies on its surface structural Fe(II) but not released Fe^2^^+^ ions. To overcome magnetic aggregation, Fe_3_O_4_ nanoparticles can be typically immobilized on functional supports to yield a controllable structure. The composite materials activate PMS/PDS with diverse active components so as to form multiple ROSs. Pi et al. obtained OBC-Fe_3_O_4_ via coating Fe_3_O_4_ nanoparticles onto oxidized biochar (OBC) with better adsorption and pollutant degradation performance than Fe_3_O_4_ and oxidized biochar [93]. A stable chemical bond was established between spherical Fe_3_O_4_ and OBC. The oxygen content of the catalyst increased after reaction, indicating that the oxygen-containing functional group as a bridge for electron transfer played an important role in the process of adsorption and degradation. Fe_3_O_4_ activated PDS to produce radicals of ^•^OH and SO_4_^•−^ [94], while the sp^2^-hybrid C atom with defective structures and ketone groups mediated electron transfer to generate ^1^O_2_. Thus multiple ROSs including SO_4_^•−^, ^•^OH and ^1^O_2_ would participate in the degradation of tetracycline. Moreover, OBC as carrier of Fe_3_O_4_ promoted the adsorption of tetracycline on the catalyst surface, thereby increasing the interaction between ROSs and tetracycline and enhancing its degradation. Liu et al. designed a core-shell iron-carbon nanocomposite catalyst (Fe@CNs as shown in Figure 4a) using sodium alginate as a template to activate PDS and degrade bisphenol A [95]. Under the protection of the carbon shell, the overall iron leaching was less than 3 μg·L^−1^ as the solution pH was 5~9, far lower than the permissible wastewater discharge standard. The carbon component not only offered larger surface area for the uniform distribution of active sites, but also acted as an excellent electron transfer carrier for PDS catalytic oxidation processes, while the incorporation of Fe enhanced redox activity of the catalyst to favor the PDS activation as evidenced by linear sweep voltammetry (LSV) tests (Figure 4b) [96]. In other similar research works using Fe_3_C/NC [97] and Fe-N/C [98], it was verified that Fe-C composite could express great synergy for catalytic PMS activation. For example, Fe-N/C could exhibit 34-fold higher reactivity than N/carbon alone towards bisphenol F degradation [98]. Based on the radical scavenging and EPR tests, ^1^O_2_ was identified as the main reactive species and coexisted with SO_4_^•−^ and ^•^OH under the catalysis of Fe-C composite. N-doped C region acted as the active center for electron transfer, and Fe affected the electronic state of the adjacent C region and increased the charge density for PMS activation, which is in consistent with the process of PDS activation [99].

Overall, the reaction between reductive Fe and PDS/PMS generally underwent radical processes involving SO_4_^•−^ and ^•^OH oxidations. The modification of Fe-based catalysts with carbon or copper materials would form different reactive sites for collaborative PDS/PMS activation. Carbon and Cu species are able to activate PDS/PMS to form ^1^O_2_, while Fe^0^ or Fe^II^ would accelerate regeneration of reactive activators. In addition to Fe/C and Fe/Cu composites, novel Fe-based glasses have attracted increasing research interest for catalytic activation of PDS/PMS. The Fe-based glasses can be easily prepared with unique atomic packing structure and present in the form of ribbons rather than powders. Zero-valent iron inside glasses could provide abundant reactive sites for peroxides activation but with much lower mass loss so as to ensure an excellent reusability [100,101]. Given the superior activity of Fe-based glasses, future efforts to tune their properties to activate PDS/PMS to produce multiple ROSs including ^1^O_2_ are highly desirable.

### 5.2. Cobalt-Based Catalysts

**Figure 5:**
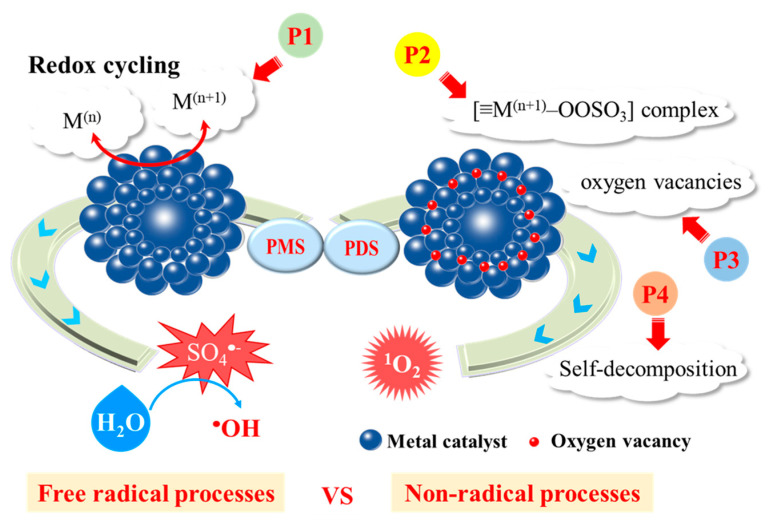
Schematic illustration of pathways of PMS/PDS activation in metal-catalyzed heterogeneous systems. P1: redox cycling; P2: formation of metal-PMS/PDS complex; P3: generation of oxygen vacancies; P4: PMS/PDS self-decomposition.

Among transition metal ions (Fe^2^^+^, Co^2^^+^, Mn^2^^+^, Ni^2^^+^), Co^2^^+^ shows the best catalytic performance for PMS activation [103]. However, excessive Co dispersed in water causes more hazardous impacts on both the environment and public health than other metal ions [64]. As an alternative, heterogeneous catalysts, especially cobalt-containing materials, such as CoOOH [104], and CoFe_2_O_4_-_x_ [105], show excellent performance in activating PMS for the generation of ^1^O_2_. Zhang et al. focused on Co-OOH nanoparticles owing to the good hydrophilic and electronic transmission rate [104]. They observed that 2,4-DCP could be completely degraded in the CoOOH/PMS system within 120 min, whereas the degradation rates for 2,4-DCP in Co_3_O_4_/PMS and CoFe_2_O_4_/PMS system were 33% and 73%, respectively. In the Co-catalyzed systems, the redox cycle of Co(III)/Co(II), as evidenced by XPS analysis, was the driving force for PDS/PMS activation as elucidated in Equations (20) and (21). Meanwhile, this redox cycling could be enhanced in the presence of the sufficient surface hydroxyl groups on CoOOH, which expedited the regeneration of CoOH^+^ intermediate to promote catalytic oxidation rate. In addition to sulfate radicals, ^1^O_2_ was produced via self-decomposition of PMS in the CoOOH/PMS system at a rate constant of 0.2 M^−1^•s^−1^ as shown in Equation (22) (P4 in Figure 5). The main cause of the ^1^O_2_ formation was attributed to the recombination of O_2_^•−^ (Equation (23)).

≡Co(III) + HSO_5_^−^ → ≡Co(II) + SO_5_^•−^ + H^+^ (20)

≡Co(II) + HSO_5_^−^ → ≡Co(III) + SO_4_^•−^ + OH^−^ (21)

HSO_5_^−^ + SO_5_^2−^ → HSO_4_^−^ + SO_4_^2−^ + ^1^O_2_ (22)

2O_2_^•−^ +2H_2_O → ^1^O_2_+ H_2_O_2_ + 2OH^−^ (23)

For the systems containing both radical and non-radical processes, some water matrices may function as the influential factor that regulates the contribution for ^1^O_2_. In a PMS activation system with Co_3_O_4_ nanowires as the catalyst, the effect of carbonate ions (CO_3_^2^^−^) was investigated for bisphenol A degradation [106]. It was revealed that ^•^OH and SO_4_^•−^ were the main ROSs in the absence of CO_3_^2^^−^ (P1 in Figure 5), but in the presence of CO_3_^2^^−^, a faster contaminant degradation rate was obtained because of the enhanced formation of ^1^O_2_, especially when the solution pH rose up to the pH_PZC_ of Co_3_O_4_. Carbonate anions could suppress Co dissolution and facilitate the conversion of catalytic center from Co(II) to Co(III), while the system switched from radical oxidation to ^1^O_2_-dominated non-radical process. These findings endorsed that the coupling of Co(III) and CO_3_^2^^−^/OH^−^ would have a synergistic effect by functioning as electron and proton acceptors, instead of a simple Co(II)/Co(III) redox cycling. The metal particles tend to aggregate in the water phase, and Co^2+^ ions would leach once the pH value is not well controlled. To overcome these limitations, nano-carbon materials, including two- or three-dimensional carbon materials, are commonly employed for metal-carbon nanocomposites fabrication. Co immobilized with carbon can offer higher catalytic efficiency for PMS or PDS activation. The co-doping strategy can not only adjust the electronic structure of the carbon catalyst, but also prevent metal leaching and simplify catalyst recovery. The immobilization of Co on carbon materials, as shown in Figure 4c, would also reduce secondary contamination of Co leaching [107]. The Co-C interaction could increase the Fermi level and chemical activity of functionalized C atoms to enhance PMS or PDS activation for pollutants degradation (Figure 4d). Several points were proposed for the reaction mechanism. First, the involvement of adsorptive carbon can facilitate the enrichment of aqueous pollutants and PMS ions to the microenvironment of internal active sites [108]. Second, the reactive Co species effectively activate PMS or PDS with production of SO_4_^•−^ and ^•^OH to achieve free radical oxidation. Third, the Co and N doping produced more defect sites and carbon graphitization, which could promote electron transfer and activate adjacent C atoms for ^1^O_2_ based non-radical degradation (Figure 4e). Moreover, the Co/Fe co-doping into plain N-C was expected to form synergistic effect for more efficient catalysis [109,110]. The existence of binary metals would accelerate redox cycling like a Fenton-like reaction. The simultaneous generation of multi-ROSs in both carbon-mediated and metal-mediated PMS/PDS activation systems facilitated deeper degradation of target pollutants (Figure 4f).

### 5.3. Manganese-Based Catalysts

Mn-associated catalysts are effective PMS/PDS activators with advantageous features like Mn being an Earth-abundant element and less toxic in nature, as compared to Co [111]. For example, a series of manganese nano catalysts with different oxidative states demonstrated potential catalysis for atrazine elimination through radical and non-radical activation of PMS [112]. α-MnO_2_ nanowires revealed higher catalytic performance due to their ability to facilitate electron transfer to maintain the redox cycle between Mn(IV) and Mn(III). In another study, both α-MnO_2_ and β-MnO_2_ (one-dimensional) displayed relatively effective PDS activation for selective mineralization of organic pollutants in wastewater [19]. Huang et al. found that ^1^O_2_ could be formed in the PMS/MnO_2_ system under acidic conditions [113]. A metastable manganese intermediate (≡MnIV−O−O−SO_3_) formed when S_2_O_8_^2^^−^ attached on the MnO_2_ surface. ≡MnIV−O−O−SO_3_ would further react with S_2_O_8_^2^^−^ to break the Mn(IV)−O bond along with the formation of O_2_^•−^. Afterward, ^1^O_2_ was generated as the primary ROSs through direct oxidation of O_2_^•−^ by Mn(IV), O_2_^•−^ recombination, and the reaction between O_2_^•−^ and metastable manganese intermediates at neutral pH (Equations (24)–(26)). Besides, Mn-doped graphite-based carbon nitride (MnCN) also provided good catalysis for PMS oxidation of acetaminophen (ACT) [114]. As indicated in the XPS spectrum, 40% of Mn existed in the Mn(III) state, while N coordinated with Mn as Mn-N. Under optimized conditions, 100% of ACT was removed within 15 min in the MnCN/PMS system. PMS would attach to Mn-N and produce superoxide anions which later transformed to ^1^O_2_. Compared to phenols and nitrobenzene, ACT exhibited significant degradation by ^1^O_2_ via attacking electron-donating acylamino groups:

2[≡Mn(IV)−OH]^III^ + HS_2_O_8_^−^ → 2[≡Mn(IV)−O−O−SO_3_]^II^ + 3H^+^ (24)

2[≡Mn(IV)−O−O−SO_3_]^II^ + 4H_2_O + S_2_O_8_^2−^ → 2[≡Mn(III)−OH]^II^ + 4SO_4_^2−^ + 2O_2_^•−^ + 8H^+^ (25)

[≡Mn(IV)−O−O−SO_3_]^II^ + O_2_^•−^ + OH^−^ → [≡Mn(III)−OH]^II^ + SO_4_^2−^ + ^1^O_2_ (26)

Oxygen vacancies could be important active site for Mn oxides (P3 in Figure 5). For instance, Jie et al. manufactured the MnO_2-x_ rattle-type microspheres that a large number of oxygen-defective MnO_2_ nanoflakes vertically arranged on the surface (OD-MnO_2-x_-RM), and altered the amount of oxygen vacancies by H_2_ reduction treatment for various treatment times (20, 40, 60 and 80 min) [115]. PMS was activated to form ^1^O_2_ due to the presence of oxygen vacancies and unique nanoarchitecture of the MnO_2-x_ rattle-type microspheres catalyst. The turnover frequency of the optimized catalyst sample OD-MnO_2-x_-RM (40 min) revealed it as the best-performing catalyst even though OD-MnO_2-x_-RM (60 min) possessed the richest oxygen vacancies.

### 5.4. Copper-Based Catalysts

Copper oxide (CuO) is considered as one of the most promising catalysts for the activation of PDS and PMS when evaluated in term of cost and availability. The activity and stability of nanocrystals is strongly dependent on orientation, dimension as well as the crystallographic structure. Du et al. found that sheet-like CuO with preferential exposed crystal facet (001) exhibited much higher reactivity toward catalytic PDS activation than spindle-like CuO [88]. The activation of PDS on CuO mainly followed a non-radical process [116]. To control the morphology and structure of CuO catalyst, Wang et al. applied polyethylene glycol as a structure directing agent [117]. They noticed that CuO-3 with controlled structure reflected better catalytic potential for PMS activation and relatively higher degradation of phenolic compounds and associated organic pollutants found in water. The complex intermediate ≡Cu(II)−(O)OSO_3_^−^ on catalyst surface was proposed to react with PMS to produce O_2_^•−^. It was verified that ^1^O_2_ rather than ^•^OH and SO_4_^•−^ was the main ROSs, and O_2_^•−^ could be an important precursor of ^1^O_2_ in the PMS/CuO-3 system. Interestingly, some reports also found that the CuO/PMS system could perform efficient for saline wastewater treatment due to the good anti-interference nature [118,119].

Another strategy refers to the use of functional support to obtain hybrid structure as well as relocate site electrons. It was reported that the Cu-O-C bond formed by immobilizing CuO on two-dimensional rGO greatly promoted catalytic PDS oxidation for trichlorophenol [88]. The confinement of rGO in hybrid material improved interfacial electron mobility between catalyst and PMS [120]. Also the Cu-O-rGO composite showed better potential to produce more oxygen vacancies for ^1^O_2_ generation. Artificial creation of oxygen vacancies effectively modulates the electronic structure of metal oxides, including CuO. This kind of modulation has been proven efficient for boosting catalytic performance [121]. Yu et al. verified that the incorporation of copper into zinc ferrite catalyst could harvest rich oxygen vacancies. The co-participation of Fe and Cu moieties contribute more active sites for catalytic PMS decomposition, and ^1^O_2_ and O_2_^•−^ were detected as the dominant ROSs. According to their results, 96.6% of ciprofloxacin (CIP) was mineralized within 15 min, and the catalyst exhibited good stability and reusability [122]. Furthermore, an easy hydrothermal-calcination route was applied to synthesize CuO-CeO_2_ composite for the activation of PMS to generate ^1^O_2_ [123]. The rate constant noted for the CuO-CeO_2_/PMS system was 7–11 times higher than that observed in other systems, such as PMS alone, or CeO_2_/PMS, and CuO/PMS systems. Better electron transfer and more oxygen vacancies reflected the synergy between CuO and CeO_2_, which contributed to remarkable ^1^O_2_ generation during PMS decomposition.

The surface structure of catalysts could be an influential factor. Jawad et al. reported that the incorporation of non-redox MgO into CuO/Fe_3_O_4_ catalyst would surprisingly enhance the catalytic performance on PMS activation, and also switch the activation mechanism from a free-radical pathway with generation of SO_4_^•−^ to ^1^O_2_-based non-radical process [124]. The Cu(II)/Cu(III) redox pair no longer acted as the catalytic center, but the incorporation of MgO facilitated the formation of deficient copper [≡Cu(III)–OH]^II^ and the enrichment of extensive ionic PMS. Then [≡Cu(III)–OH]^II^ reacted with PMS to form [≡Cu(III)–OOSO_3_] complex (Equation (27), P2 in Figure 5). In other cases, divalent copper complex in form of [≡Cu(II)–OOSO_3_] acted as this vital intermediate [120]. The electron transfer from SO_5_^2^^−^ to ≡Cu sites ultimately demonstrated the decomposition of [≡Cu–OOSO_3_] to form O_2_^•−^ as a precursor of ^1^O_2_ (Equation (28)):

[≡Cu(III)−OH]^II^ + HSO_5_^−^ → [≡Cu(III)−(O)OSO_3_^−^]^I^ + H2O (27)

2[≡Cu(III)−(O)OSO_3_]^I^ + HSO_5_^−^ +3H_2_O → 2[≡Cu(II)−OH]^I^ + 2O_2_^•^^−^ + 3SO_4_^2^^−^ + 7H^+^ (28)

### 5.5. Other Metallic Catalysts

Addtionally, metal oxides of perovskites (ABO_3_ structure) [8] and spinel (AB_2_O_4_ structure) [125] have attracted increasing interest due to their high stability and strong oxidation potential. At octahedral and tetrahedral sites, different types of cations with similar values of crystal field stabilization energies can substitute the metal situating in crystal lattice and form partial oxygen defects for the regulation of the band structure and the recycle of redox pairs [126]. The oxygen vacancies on the surface of metal/metal oxide play an important role in the generation of ^1^O_2_ and efficient activation of PMS. The surface and lattice oxygen vacancies are expected to facilitate oxygen adsorption and storage, and accelerate oxygen mobility, which are important for rapid generation of O_2_^•−^ and their following conversion to ^1^O_2_ [105,127]. Gao et al. prepared LaBO_3_ perovskites with different B site metal (B= Fe, Zn, Mn and Ni) to investigate the effect of B site metals on the PMS activation and ^1^O_2_ generation route [8]. It was observed that as high as 21.8% of oxygen defects was monitored for LaNiO_3_. Ofloxacin (OFX) was completely degraded by the LaNiO_3_/PMS system, which could be assigned to the effect of oxygen defects on ^1^O_2_ generation. The surface oxygen defects of perovskite could lower the energy barrier of spontaneous PMS decomposition on LaBO_3_ surface, which is an important pathway for the formation of ^1^O_2_. Chen et al. also suggested that the cobalt ions in the tetrahedral sites were inclined to be substituted by manganese ions with larger ionic radius [128] accompanied by the generation of vacancies on the O sites [125]. Meanwhile, some active oxygen might react with HSO_5_^−^ to produce ^1^O_2_. More oxygen vacancies would facilitate interfacial electron transfer of PMS activation [129,130].

In addition to the usual transition metals, noble metals also show potential capability to activate PMS with generation of ^1^O_2_ for the degradation of selective organic pollutants. Ahn et al. found that noble metals including Pt, Pd, Au, and Ag immobilized on Al_2_O_3_ or TiO_2_ could mediate electron transfer from organics to PMS to achieve non- radical oxidation [131]. The catalytic performance exhibited a dependency on the type of noble metal in an order of Pd > Pt ≈ Au ≫ Ag. To further understand the intrinsic catalytic mechanism, Wang et al. anchored Pd particles in the cavity of g-C_3_N_4_ as a heterogeneous catalyst (Pd/g-C_3_N_4_) to activate PMS with generation of ^1^O_2_ and O_2_^•−^ for bisphenol A degradation [132]. Noteworthily, less than 10% of bisphenol A could be removed by g-C_3_N_4_/PMS alone, while 91% of bisphenol A could be degraded in 60 min by Pd/g-C_3_N_4_/PMS. However, it was observed that Pd^0^ might convert to Pd^(II^^)^ as indicated by the XPS results that the Pd^0^/Pd^II^ ratio would decrease from 2.02 to 1.19 after the reaction (Equations (29)–(34)). The catalytic ability was significantly influenced by solution pH and reached maximum at pH 9 because ^1^O_2_ would attack deprotonated organic compounds at a higher oxidation rate compared to undissociated ones. The mechanism involves the following points: (i) H_2_O_2_ and Pd^0^·OH formed by the reaction between HSO_5_^−^ and H_2_O under catalysis of Pd^0^ (Equations (29)–(30)); (ii) the disassociation of Pd^0^·OH generates ^1^O_2_ according to Equations (31) and (32); (iii) PMS was catalyzed by Pd^0^ into intermediate ^•^OHPd^0^SO_4_^•−^ (Equation (33)), which was then decomposed into Pd^II^, SO_4_^2^^−^ and H^+^ (Equation (34)):

HSO_5_^−^ + H_2_O → H_2_O_2_ + HSO_4_^−^ (29)

2Pd^0^ + H_2_O_2_ → 2Pd^0^·OH (30)

2Pd^0^·OH → H_2_O + Pd^0^·O + Pd^0^ (31)

2Pd^0^·O → 2Pd^0^ + ^1^O_2_ (32)

HSO_5_^−^ + Pd^0^ → ^•^OHPd^0^SO_4_^•−^ (33)

^•^OHPd^0^SO_4_^•−^ + H_2_O →···→ Pd^II^ + SO_4_^2−^ + 2H^+^ (34)

## 6. Implications for In Situ Applications and Future Perspectives

### 6.1. Implications for In Situ Applications

The wide occurrence of emerging organic contaminants, such as personal care products and pharmaceuticals (PCPPs), endocrine disrupting chemicals (EDCs), pesticides and surfactants, in natural environment has forced rapid development of PDS/PMS-based AOPs for in situ environmental remediation. Efficiency of conventional PDS/PMS oxidation processes is usually affected by practical matrix conditions, such as temperature, solution pH and salinity. Typically, it is generally recognized that high salinity is a big roadblock for the degradation of organic contaminants in AOPs. Radicals of ^•^OH and SO_4_^•−^ can easily reacted with Cl^−^, NO_3_^−^ to form corresponding byproducts of Cl^•^ and NO_3_^•^, and even suppressed in the existence of carbonate and phosphate. This inhibition under high salinity seems to be greatly weakened during non-radical AOPs [118]. An efficient destruction of bisphenol A in high salinity water was observed during ^1^O_2_-dominated PMS activation by using nitrogen-doped carbon as the catalyst [133]. Anions including Cl^−^, NO_3_^−^, HCO_3_^−^, H_2_PO_4_^−^ even in concentrations up to 500 mM exhibited insignificant effects on bisphenol A degradation. This insensitivity to the water matrix is related to the unstable nature of PMS. The unsymmetrical PMS easily undergo self-decomposition under nucleophilic attack by high dose of Cl^−^, HCO_3_^−^, and H_2_PO_4_^−^ with production of ^1^O_2_. Unlike SO_4_^•−^ and ^•^OH, ^1^O_2_ is a moderate oxidant that unable to oxidize these anions to anion-derived radicals. Besides, soil nature organic matter (NOM) is a complex factor for PMS/PDS activation. SO_4_^•−^ and ^•^OH radicals are likely to oxidize these background organic constituents so that displaying suppression for target pollutants degradation, but NOM with abundant quinone or semiquinone groups is also a potent PMS activator in alkaline conditions as indicated in Section 3. Moreover, NOM in aquatic systems commonly acts as photosensitizer for ^1^O_2_ formation rather than quencher [26], so the negative effect of NOM in ^1^O_2_-dominated system might be marginally limited [19,91]. A bench column study by Yang et al. showed that HCO_3_^−^ and Cl^−^ did not show detrimental effects on TCE degradation and the effect of NOM were negligible at high PMS dosage during in situ chemical oxidation of trichloroethylene (TCE) with bimetallic Fe-Mn oxide as the catalyst [134]. Their EPR and radical scavenging results implied that SO_4_^•−^, ^•^OH and ^1^O_2_ contributed to TCE degradation. Involvement of various highly ROSs during AOPs resulted in high rate of TCE degradation and dichlorination compared to conventional H_2_O_2_-based in situ oxidation. Besides, solution pH is another influential parameter depending on characteristics of activator. In homogeneous activation systems, ^1^O_2_ could be directly generated via PDS/PMS activation under neutral (6.5 ± 0.3) and alkaline condition because the surface hydroxyl groups could improve the chemical binding with PDS/PMS. In heterogeneous system, metal could activate PMS with formation of ^1^O_2_ in a broader pH range [91]. The nature of catalyst structure, singlet oxygenation and electron transfer are crucial factors behind the formation of ^1^O_2_ [90].

In addition, the impact of subsurface minerals on PDS/PMS-based in situ oxidation cannot be ignored. Studies by Zhu et al. indicated that PDS interacting with different crystalline MnO_2_ forms would transform to ^1^O_2_ as the reactive species for phenol abatement. Ahmad et al. and Yu et al. found that synthetic birnessite (manganese oxide) and goethite (iron oxide) were effective mineral for both PDS and PMS activation during in situ chemical oxidation [135,136]. Non-radical pathways accounted for oxidation in birnessite and goethite catalytic PMS systems, and this process could be promoted in presence of soil organic matter. It was concluded that the PDS/PMS decomposition mostly relied on the nature of the mineral surface as well as the rate of metal dissolution. Once persulfate was injected into the contaminated plume, its decomposition occurred due to frequent interaction with aquifer materials including soil organic matter and minerals. Sra et al. verified that the injection of unactivated persulfate into a gasoline source zone could abate a maximum of 46%–86% of gasoline contaminants after two months of remediation [137].

### 6.2. Future Perspectives

Despite the fact PDS/PMS-based AOPs with production of ^1^O_2_ have shown interesting properties in bench scale studies, there are several issues deserve further scientific investigations.

First, except for NaN_3_, FFA, more suitable quenchers and quantitative methods are needed to further prove key role of singlet oxygen in the rapid degradation of pollutants.

Second, although MFCMs were recognized as desirable potential catalysts, discrepant catalytic activity has been obtained due to different material configuration and surface functional groups. Further studies have to be carried out to figure out the effect of MFCMs characteristics on the reaction efficiency and its key relationship with ROSs production.

Third, the adoption of PDS/PMS-based AOPs for full scale applications largely relies on high pollutant degradation efficacy and cost-effective catalysts. Thus, a novel cheap and stable catalyst which can activate PDS/PMS to exploit multiple oxidation pathways would be truly desirable. In general, ^1^O_2_ is recognized to be more selective to mildly oxidize electron-rich substrates and shows much stronger anti-interference capability towards inorganic ions as well as natural organic matters. Therefore, ^1^O_2_ can be properly used for disinfection of pathogenic bacteria. By contrast, SO_4_^•−^ and ^•^OH exhibit more powerful oxidation ability but poor resistance to background water impurities because of radical quenching. Therefore, degradation via multiple oxidation pathways involving different ROSs, such as SO_4_^•−^, O_2_^•−^, ^•^OH and ^1^O_2_ in the same reaction system, is expected to reach a higher oxidation efficiency, especially for wastewaters containing antibiotics and antibiotic resistant bacteria. Finally, the novel techniques available for PDS activation are still limited as compared to PMS-based AOPs. It is well-known that the commercial PDS is cheaper than PMS, and efficient PDS-based AOPs are expected to produce less sulfate ion than PMS-based AOPs after reactions since PMS only constitutes 1/3 of Oxone^®^. It is thus crucial to develop more PDS-based catalysts with efficient generation of different ROSs for the sake of future commercialization.

## 7. Conclusions

This paper presents an overview of ^1^O_2_ formation via non-radical activation of PDS/PMS in both homogenous and heterogeneous reaction systems. In homogeneous systems, ketones, quinones and alkaline are effective to activate PMS to generate ^1^O_2_ while PDS is more likely to be decomposed with generation of radicals. For heterogeneous systems, MFCMs including CNTs, reduced GO and biochar materials have received much attentions. N-doping and structure tailoring endow MFCMs with more lattice vacancies and defect sites for the exploitation of PDS/PMS. In addition, ketone functional groups are able to provide additionally accessible active sites for MFCMs, and the catalytic efficiency could be significantly tuned by controlling the number of ketone groups. Furthermore, the effectiveness of transition metals such as Co, Cu and Mn were discussed in regard of activating PDS or PMS to initiate ^1^O_2_ production under some specific conditions. Surface complexation and redox reactions were proposed as the main mechanisms for metal-mediated activation. Additionally, composite catalysts with multiple functions were discussed. Metal would be doped or immobilized into carbon and membrane to show synergistic effect with less metal leaching, enhanced catalytic stability and reusability. Overall, ^1^O_2_ can be formed either as the main ROSs to dominate the oxidative degradation or co-exist with radicals including SO_4_^•−^, O_2_^•−^ and ^•^OH. It is largely evidenced that catalysts, oxidant type, reaction parameters are all influential factors for ^1^O_2_ production.

## Figures and Tables

**Table 1:** Performance and mechanism for PDS/PMS oxidation of pollutants in typical homogenous systems.

Systems	Target/Pollutant	Degradation Rate and Time	Radicals Involve	Active Catalytic SitesOr Activation Mechanism	References
phenols/PMS	sulfamethoxazole	100% in 60 min at pH 10	^1^O_2_	quinone intermediates formed from phenolic parents	Zhou et al. [34]
PMS	chlorophenols	65% of 4-CP, 70% of 2,4-DCP and 95% of 2,4,6-TCP in 60 min at pH 9	^1^O_2_, SO_4_^•−^, ^•^OH	chlorophenols	Li et al. [35]
ketones/PMS	/	/	^1^O_2_,	ketone carbonyl	Lange and Brauer [40]
benzoquinone/PMS	sulfamethoxazole	86% in 3 min at pH 10	^1^O_2_	quinone carbonyl	Zhou et al. [41]
benzoquinone/PMS	/	/	^1^O_2_	quinone carbonyl	Zhang et al. [43]
Cl^−^/phenol/PMS	phenols	100% in 60 min at pH 10	^1^O_2_	Cl^−^ interact with PMS to form chlorophenols and benzoquinone	Li et al. [44]
base/PMS	acid orange 7	90% in 60 min	^1^O_2_, O_2_^•−^	O_2_^•−^ intermediates for ^1^O_2_ generation under too much pH conditions	Qi et al. [45]
base/polyphosphates/PMS	acid orange 7	100% in 500 s	^1^O_2_, O_2_^•−^	remains unclear	Luo et al. [47]
BO_2_^−^/PMS	acid red 1	97.8% in 10 min	^1^O_2_	nucleophilic mechanisms	Rao et al. [48]
CO_3_^2−^/PMS	acid orange 7	100% in 40 min	^1^O_2_, O_2_^•−^	PMS self-decomposition, O_2_^•−^ intermediates by base-catalyzed hydrolysis for ^1^O_2_ generation	Nie et al. [49]
Cl^−^/PMS	2,4-dimethylphenol	100% in 20 min	SO_4_^•−^, ^•^OH, ^1^O_2_, Cl^•^	/	Wang et al. [50]
Cl^−^/PMS	methylene blue	100% in 120 min	^1^O_2_, Cl^•^	PMS self-decomposition, reactive chlorine	Yang et al. [51]

**Table 2:** Performance and mechanism of typical MFCMs catalysts for PDS/PMS oxidation of pollutants.

Catalyst	Oxidant	Target Pollutant	Degradation Rate and Time		Radicals Mechanism	Active Catalytic Sites	References
reduced graphene oxide	PMS	phenol	100%	45 min	^1^O_2_	sp^2^ carbon, defective edges	Duan et al. [53]
single-wall carbon nanotubes	PDS	4-chlorophenol	100%	60 min	^1^O_2_	sp^2^ carbon, nonradical electron-transfer	Yun et al. [58]
carbon nanotubes	PDS	phenol	100%	30 min	/	nonradical electron-transfer	Ren et al. [59]
carbon nanotubes	PDS	2,4-dichlorophenol	95.9%	30 min	^1^O_2_, O_2_^•−^	sp^2^ carbon	Cheng et al. [60]
nitrogen-doped carbon nanotubes	PMS	phenol	95.6%	10 min	/	nonradical electron-transfer	Ren et al. [62]
N-doping reduced graphene oxide	PMS	Sulfachloro-pyridazine	100%	9 min	SO_4_^•−^, ^•^OH, ^1^O_2_	hydroxyl group, sp^2^ carbon, pyridine N, pyrrole N, graphite N	Kang et al. [67]
N-S co-doped graphene	PMS	methyl *p*-hydroxy-benzoate	100%	30 min	SO_4_^•−^, ^•^OH, ^1^O_2_	sp^2^ carbon, pyrrolic N, pyridinic N, graphitic N, thiophenic S	Sun et al. [69]
amino-functionalized mesoporous silica anchoring N-doped graphene oxide	PMS	bisphenol A	100%	600 min	^1^O_2_	sp^2^ carbon, phenolic hydroxyl group, amino group	Zhang et al. [73]
sludge-biochar (600 °C)	PMS	bisphenol	100%	30 min	^1^O_2_	ketone structure inside the biochar	Huang et al. [76]
biochar doped with N and S	PMS	metolachlor	about 80%	120 min	^•^OH, ^1^O_2_	surface ketone of biochar	Ding et al. [77]
porous nitrogen-doped reduced graphene oxide	PMS	phenol	100%	25 min	^•^OH, ^1^O_2_, O_2_^•−^	structure vacancies with two nitrogen atoms of graphene structure	Wu et al. [78]
sludge-derived biochar	PDS	sulfamethoxazole	94.6%	180 min	^1^O_2_	sp^2^ carbon, Fe(II), N atoms	Yin et al. [79]
nitrogen-doped sludge carbon	PMS	methylene blue	98.7%	20 min	SO_4_^•−^, ^•^OH, ^1^O_2_	not mentioned	Hu et al. [81]
graphited nanodiamond	PDS	phenol	100%	10 min	SO_4_^•−^, ^•^OH, ^1^O_2_	nonradical electron-transfer	Lee et al. [82]
nano diamonds with a core/shell structure	PMS	phenol	100%	90 min	/	sp^2^/sp^3^ carbon of graphite structure	Duan er al. [83]
porous carbon aerogel	PDS	rhodamine B	100%	60 min	^1^O_2_, O_2_^•−^	sp^2^ carbon, defective edges and the carbon edges of carbon aerogel	Jiang et al. [85]
N-doped chitosan-derived carbon nanosheets	PMS	sulfacetamide	100%	10 min	^1^O_2_, O_2_^•−^	sp^2^ carbon, graphitic N	Chen et al. [86]

**Table 3:** Performance and mechanism of typical heterogeneous metallic catalysts for PDS/PMS oxidation of pollutants.

Oxidant	Catalyst	Target/Pollutant	Degradation Rate and Time		Radicals Involve	Active Catalytic SitesOr Activation Mechanism	References
PDS	β-MnO_2_	phenol	over 99%	180 min	^1^O_2_, O_2_^•−^	metastable manganese intermediates for O_2_^•−^ generation	Zhu et al. [19]
	sheet-like CuO	2,4,6-trichlorophenol	90%	180 min	^•^OH, non-radical	facet (001) of CuO, electron-defective center	Du et al. [88]
	CoFe_2_O_4-x_	bisphenol A	98%	60 min	SO_4_^•−^, ^•^OH, ^1^O_2_	Fe(III)/Fe(II), Co(III)/Co(II), oxygen vacancies,	Wu et al. [105]
	CuO	ciprofloxacin	100%	30 min	^1^O_2_, O_2_^•−^, SO_4_^•− •^OH	Cu(II)/Cu(III) for O_2_^•−^ and ^1^O_2_, Cu(I)/Cu(II) for SO_4_^•−^, ^•^OH	Xing et al. [116]
PMS	LaNiO_3_	ofloxacin	93%	10 min	^1^O_2_, SO_4_^•−^, ^•^OH	Ni(III)/Ni(II), oxygen vacancies	Gao et al. [8]
	nZVI/Cu^2+^	rhodamine B	99.3%	60 min	^1^O_2_, O_2_^•−^, SO_4_^•−^, ^•^OH	Fe(III)/Fe(II), Cu(II)/Cu(I)	Li et al. [90]
	Fe^0^-Mt	bisphenol A	99.3% at pH 3	120 min	^1^O_2_, O_2_^•−^, SO_4_^•−^, ^•^OH	Fe^0^, released Fe^2+^	Yang et al. [91]
	CoOOH	2,4-dichlorophenol	100%	120 min	^1^O_2_, O_2_^•−^, SO_4_^•− •^OH	Co(III)/Co(II), -OH	Zhang et al. [104]
	Co_3_O_4_/CO_3_^2−^	bisphenol A	100%	10 min	^1^O_2_, O_2_^•−^, SO_4_^•− •^OH	Co(III)/Co(II), OH^−^, CO_3_^2−^	Zhu et al. [106]
	Mn oxides in different structure	atrazine	100%	100 min	^1^O_2_, SO_4_^•−^, ^•^OH	Mn(IV)/Mn(III), Mn(III)/Mn(II)	Zeng et al. [112]
	δ-MnO_2_	bisphenol A	42%	10 min	^1^O_2_, SO_4_^•−^, ^•^OH	δ-MnO_2_ direct oxidation, Mn(IV)/Mn(III)	Huang et al. [113]
	Mn-g-C_3_N_4_	acetaminophen	100%	15 min	^1^O_2_, O_2_^•−^	Mn(III)/Mn(II) in the N-pot	Fan et al. [114]
	Oxygen-defective MnO_2_	bisphenol A	100%	60 min	^1^O_2_, SO_4_^•−^, ^•^OH	oxygen vacancies	Yu et al. [115]
	CuO	bisphenol A	100%	60 min	^1^O_2_, O_2_^•−^	Cu(II)-(O)-OSO_3_^−^ formed on surface of CuO for O_2_^•−^ generation	Wang et al. [117]
	magnetic CuO nanosheet	AO7 in high salinity wastewater	95.8%	30 min	^1^O_2_, SO_4_^•−^, ^•^OH	synergistic effect of Cu(I)/Cu(II) and Fe(II)/Fe(III)	Li et al. [118]
	copper substituted zinc ferrate	ciprofloxacin	96.6%	15 min	^1^O_2_, O_2_^•−^, SO_4_^•−^, ^•^OH	Fe(III)/Fe(II), Cu(II)/ Cu(I), oxygen vacancies	Yu et al. [122]
	CuO-CeO_2_	rhodamine B	100%	60 min	^1^O_2_, O_2_^•−^, SO_4_^•−^, ^•^OH	Ce(IV)/Ce(III), Cu(II)/Cu(I), oxygen vacancies, electron transfer	Li et al. [123]
	CuOMgO/Fe_3_O_4_	4-chlorophenol	100%	40 min	^1^O_2_, O_2_^•−^	[≡Cu^(III)^–OOSO_3_] intermediates for O_2_^•−^ generation	Jawad et al. [124]
	Co_2_Mn_1_O_4_	triclosan phenol	96.4%	30 min	^1^O_2_, SO_4_^•−^	oxygen vacancies, Co(II)/Co(III), Mn(III)/Mn(II)/Mn(IV)	Chen et al. [125]
	LaCo_0.4_Cu_0.6_O_3_	phenol	100%	12 min	^1^O_2_, SO_4_^•−^, ^•^OH	Co(II)/Co(III), Cu(II)/Cu(I), oxygen vacancies	Lu et al. [130]

**Table 4:** Performance and mechanism of metal-carbon nanocomposite catalysts for PDS/PMS oxidation of pollutants.

Oxidant	Catalyst	Target/Pollutant	Degradation Rate and Time		Radicals Involve	Active Catalytic SitesOr Activation Mechanism	References
PDS	oxidation biochar supported magnetite particles	tetracycline	92.3%	120 min	SO_4_^•−^, ^•^OH, ^1^O_2_	Fe(II)/Fe(III), sp2-hybrid C atom with defective structures, ketone groups mediated electron transfer	Pi et al. [93]
	Nuclear-shell structure iron-carbon	bisphenol A	96%	30 min	SO_4_^•−^, ^•^OH, ^1^O_2_, O_2_^•−^	Fe^0^ Fe(II)/Fe(III), electron transfer for O_2_^•−^ formation to produce ^1^O_2_	Liu et al. [95]
PMS	sludge-derived magnetic Fe^0^/Fe_3_C	ciprofloxacin	99%	20 min	SO_4_^•−^, ^•^OH, ^1^O_2_, O_2_^•−^	Fe^0^, Fe(II)/Fe(III), electron transfer for O_2_^•−^ formation to produce ^1^O_2_	Zhu et al. [96]
	Fe_3_C embedded on carbon	ibuprofen	100%	30 min	SO_4_^•−^, ^•^OH, ^1^O_2_	Fe(II)/Fe(III), N-doped carbon area, enhanced electron transfer process due to the carbon shell	Zhang et al. [97]
	iron and nitrogen co-doped porous carbon	bisphenol F	97.1%	90 min	SO_4_^•−^, ^•^OH, ^1^O_2_, O_2_^•−^	pyridine N, graphite N, adjacent C region of Fe-doping	Wu et al. [98]
	N-doped porous carbon embedded with CoO nanodots	chlorophenol	100%	30 min	SO_4_^•−^, ^•^OH, ^1^O_2_	Co(II)/Co(III), increase defects sites of C by CoO doping, enhanced electron transfer by N doping	Xie et al. [102]
	core-shell Co@C nanoparticles with nitrogen and sulfur	*p*-hydroxybenzoic acid	100%	45 min	SO_4_^•−^, ^•^OH, ^1^O_2_	sp^2^ carbon, defect sites procuced by Co and N doping, Co(II)/Co(III)	Tian et al. [107]
	CoFe alloy nanoparticles encapsulated in nitrogen doped graphitic carbon	norfloxacin	94.4%	20 min	SO_4_^•−^, ^•^OH, ^1^O_2_	Co(II)/Co(III), Fe(II)/Fe(III), neighboring C atoms of graphitic N, self-decomposition of PMS	Ding et al. [109]
	carbon-based Fe-Co oxide derived from Prussian blue	4-aminobenzoic acid ethyl ester	95.5%	60 min	SO_4_^•−^, ^•^OH, ^1^O_2_	Co(II)/Co(III), Fe(II)/Fe(III), sp^2^ hybridized carbon, pyridinic-N and pyrrolic-N	Liu et al. [110]
	Pd nanoparticles anchored C_3_N_4_	bisphenol A	91%	60 min	SO_4_^•−^, ^•^OH, ^1^O_2_	Pd^0^/Pd(II), electron transfer for ^1^O_2_ production	Wang et al. [132]
